# Breast Metastasis from Esophagogastric Junction Cancer: A Case Report

**DOI:** 10.1155/2014/489427

**Published:** 2014-06-11

**Authors:** Sanghamitra Jena, Samir Bhattacharya, Arnab Gupta, Shravasti Roy, Neetesh Kumar Sinha

**Affiliations:** ^1^Department of Surgical Oncology, Saroj Gupta Cancer Centre and Research Institute, Mahatma Gandhi Road, Thakurpukur, Kolkata 700063, India; ^2^Department of Pathology, Saroj Gupta Cancer Centre and Research Institute, Mahatma Gandhi Road, Thakurpukur, Kolkata 700063, India; ^3^Department of Cancer Surgery, VMMC and Safdarjung Hospital, New Delhi 110029, India

## Abstract

Metastasis to breast from nonmammary malignancy is only about 1.3–2.7%. A few cases of squamous cell carcinoma of esophagus and adenocarcinoma of stomach metastasizing to breast have been reported, but this is probably the first report of breast metastasis from esophagogastric junction (EGJ) cancer in the English literature. Herein we report a case of a 32-year-old patient diagnosed as adenocarcinoma of gastroesophageal junction, presenting with left breast metastasis two years after treatment. Given unusual site of metastasis in a follow-up case of EGJ cancer, not only it is challenging to differentiate it from primary carcinoma of breast but also it is important from treatment point of view. In our case, clinical data, radiology, histopathology, and immunohistochemistry (IHC) led us to reach the diagnosis.

## 1. Introduction

Stomach cancer is the fourth most common malignancy in the world, after cancers of the lung, breast, and colorectum, and is the second leading cause of cancer death in both sexes worldwide [1]. Esophageal cancer (EC) is the eighth most common cancer worldwide and the sixth most common cause of death from cancer [1]. This cancer commonly metastasizes to the lung, skeletal system, and liver [2, 3]. The sites of metastasis of EGJ cancer had not been extensively studied separately until the differentiation of esophagogastric junction cancer as a different entity from stomach and esophageal cancer came up with Siewert and Stain classification [4]. There are reported cases of adenocarcinoma of EGJ metastases to gingiva [5] and meninges [6], but even after extensive literature search for 30 years through Pubmed and Medline, we could not find any case report of breast metastases from EGJ cancer so far. Herein we report a case of a 32-year-old patient, previously diagnosed as adenoca rcinoma of gastroesophageal junction, presenting with left breast metastasis two years after completion of EGJ cancer specific treatment.

## 2. Case History

A 32-year-old female presented in September 2010, with a history of dysphagia to solid food for 2 months. Upper GI endoscopy showed an ulceroproliferative growth producing narrowing of lumen involving lower esophagus 36 cm onwards from incisor teeth. Scope could not be passed beyond the lesion. Biopsy revealed poorly differentiated adenocarcinoma. CT scan thorax and abdomen showed oval shaped hypodense area at cardioesophageal junction, projecting within lumen of esophagus.

**Figure 1:**
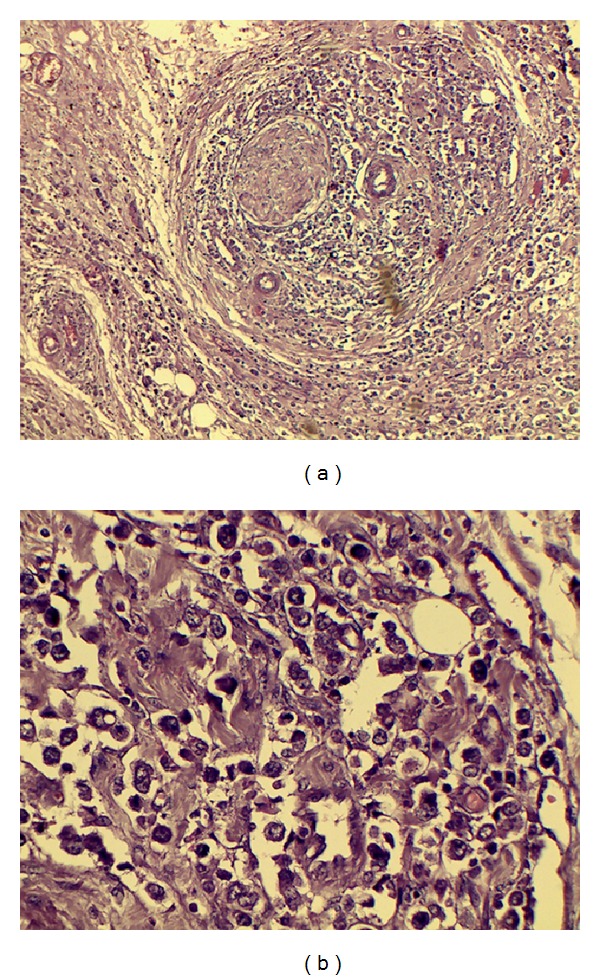
(a) Esophagogastric junction histological sample (haematoxylin-eosin stain 10x). (b) Esophagogastric junction histological sample (haematoxylin-eosin stain 40x).

The patient underwent transthoracic esophagogastrectomy with 5 cm tumor free margins. The surgery was performed by upper midline abdominal incision and right sided thoracotomy. Intrathoracic esophagogastric anastomosis was performed by circular stapler. There was a 7 cm × 5 cm growth within GE junction extending to lesser curvature. Histopathological diagnosis was poorly differentiated adenocarcinoma (Figures 1(a) and 1(b)). The tumor invaded serosal layer of stomach and one out of 10 perigastric lymph nodes was positive for metastasis. Surgical margins were negative. The AJCC classification of primary tumor was T3N1M0. After surgery, this patient received a combination chemotherapy of epirubicin (50 mg), cisplatin (250 mg), and fluorouracil (650 mg) for six cycles. Subsequently, the patient was followed up regularly.

**Figure 2:**
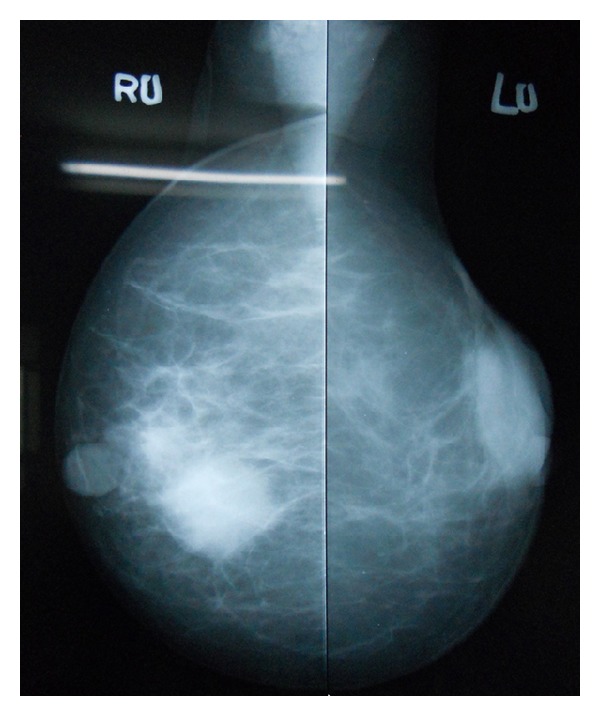
Mammography of bilateral breast.

**Figure 3:**
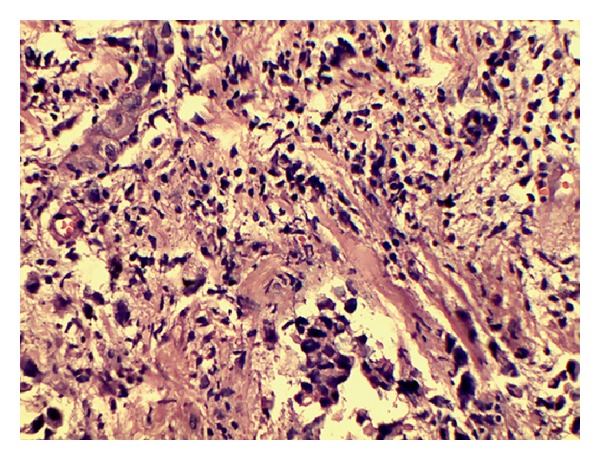
Left breast histological sample (haematoxylin-eosin stain 40x).

**Figure 4:**
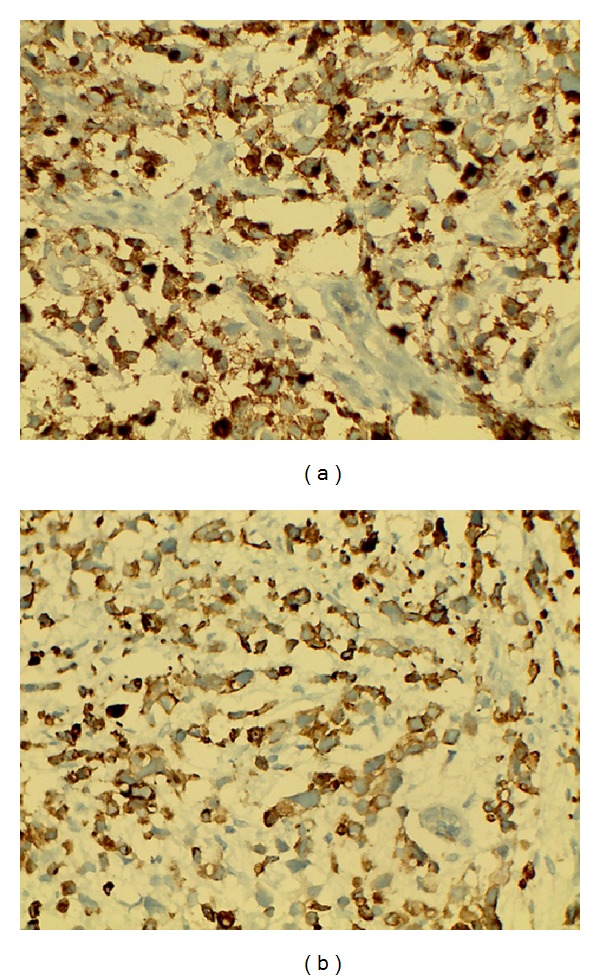
(a) Metastatic cells positive for CEA (40x). (b) Metastatic cells positive for CK 20 (40x).

Two years later, she presented with bilateral breast lumps and dysphagia. On examination, a 3 cm × 2 cm hard mobile lump was palpable in central quadrant of right breast and a 2 cm × 2 cm hard mobile lump was palpable in upper outer quadrant of left breast, without any involvement of axillary or supraclavicular lymph node on either side. In mammography, a round shaped soft tissue opacity was seen in the upper and outer region of left breast and an oval shaped soft tissue opacity in the retroareolar region of right breast (Figure 2). Trucut biopsy of right breast lump was benign, but left breast showed neoplastic cells in diffuse sheets. The cells had vacuolated cytoplasm and pleomorphic hyperchromatic nuclei. The neoplastic cells were infiltrating into breast parenchyma and destroying terminal duct-lobular unit (Figure 3). Immunohistochemistry showed that ER, PR, and Her-2-neu were negative, CEA and CK-20 were positive, and CK-7 was negative (Figures 4(a) and 4(b)). Endoscopy revealed stricture at 30 cm extending up to 38 cm. The stricture involved the distal end of the esophagus and proximal end of the gastric tube. Esophageal dilatation was done and biopsy was taken. Biopsy showed features of poorly differentiated adenocarcinoma. In view of poor general condition and progressive disease, only esophageal stenting and palliative treatment were offered. But the patient did not agree on esophageal stenting and did not turn up further.

## 3. Discussion

Metastases of gastric cancer may be found at the time of diagnosis or at some intervals after gastrectomy [7]. About 18% of patients with gastric cancer will eventually develop metastasis after gastrectomy [7]. Similarly distant metastasis occurs in 26% of locally advanced ECs in the first 2 years of initial therapy [3]. Common metastatic sites of gastric and esophageal cancer are regional lymph nodes, liver, lungs, and bone [2, 3, 8]. However, the sites of metastases from gastroesophageal junction adenocarcinoma have not been separately mentioned as the distinction of EGJ cancer from stomach and esophagus had come up with Siewert and Stain classification in 1998 [4].

Metastasis to breast by nonmammary malignancy is approximately 1.3–2.7% [9]. Although virtually all malignancies may metastasize to the breast, the most common primary tumors in decreasing order of frequency are lymphomas (17%), melanomas (15%), rhabdomyosarcomas (12%), lung tumors (8%), ovarian tumors (8%), renal cell tumors (5%), leukemia (4%), thyroid/cervical tumors (4%), intestinal carcinoid tumors (3%), squamous cell carcinomas of head and neck (3%), and leiomyosarcomas (2%) [10, 11]. In the literature only approximately 300 cases of tumor metastases to the breast have been described. Metastasis to breast arising from the gastrointestinal tract is rare [12] and only in few cases of squamous cell carcinoma of esophagus [13–15] and carcinoma of stomach [12, 16, 17] metastasis to breast has been reported. Interestingly this is the first case of adenocarcinoma of esophagogastric junction where metastasis to breast is reported in the English literature.

Metastasis to breast in a follow-up case of carcinoma EGJ is challenging to differentiate it from primary carcinoma of breast which is a more common cancer in females. Metastatic lesions however present some features that make them distinguishable from primary breast tumors. Most cases of mammary metastases were reported in younger age than primary breast cancer cases [18]. High blood flow of breast in young adults is considered as the main reason behind this. Metastasis is usually a single, painless, mobile, ipsilateral, and well-circumscribed tumor sited in the upper quadrants of the breast. Multiple, diffuse, and bilateral involvement is rare as also is the involvement of the axillary lymph nodes [16, 17, 19]. In this patient, although two lumps (one in each breast) were palpable, only one of them was found to be malignant. It was mobile, well-defined, and located in upper outer quadrant of the left breast without any axillary or supraclavicular lymphadenopathy.

Radiographically, mammographic evaluation can provide additional information. Metastatic tumors to the breast more frequently present as well-circumscribed, noncalcified dense masses. They generally lack spiculation and microcalcifications as well as architectural distortion and other skin changes [7, 8, 12, 20, 21]. This patient also had similar findings in mammography.

The histopathological features suggestive of metastases in the breast include absence of in situ carcinoma, which characterises the majority of primary breast cancers. The histological picture usually resembles the extramammary primary tumour and is not typical of breast carcinomas.

Metastases from gastric adenocarcinomas to the breast, on IHC, are usually positive for CEA and CK 20 and negative for ER, PR, and CK 7 [22, 23]. By combining the results of CEA, CK 7, CK 20, and ER and PR staining, the metastases to the breast from a primary lesion in the gastrointestinal tract could be confirmed in this study.

## 4. Conclusion

This is the first case of breast metastasis from adenocarcinoma of esophagogastric junction reported in the English literature. This has occurred in a young female two years after completion of treatment. Since breast metastasis arising from the gastrointestinal tract is rare and primary breast cancer is the second most common cancer in females in developing countries, the diagnosis may not be straight forward. However, the clinical presentation, radiological features, histopathology, and IHC study can help us in differentiating between the two conditions and deciding about further management.
